# Supplementary material for: Modeling glioblastoma heterogeneity as a dynamic network of cell states
Source: Mol Syst Biol. 2021 Sep 16;17(9):e10105. doi: 10.15252/msb.202010105 (PMC8444284; doi:10.15252/msb.202010105)
Supplement: Supplementary file 5 — Source Data for Figure 3 [file MSB-17-e10105-s001.zip › Figure3A_sourcedata/GSEA_3065/nosigdb_state4.GseaPreranked.1622718430994/RICHARDS_NOWAKOWSKI_SCIENCE_2017_MGE_IPC2_UPREG.html]

Details for gene set RICHARDS\_NOWAKOWSKI\_SCIENCE\_2017\_MGE\_IPC2\_UPREG[GSEA]

|  || Dataset | state4 |
| Phenotype | NoPhenotypeAvailable |
| Upregulated in class | na\_neg |
| GeneSet | RICHARDS\_NOWAKOWSKI\_SCIENCE\_2017\_MGE\_IPC2\_UPREG |
| Enrichment Score (ES) | -0.77266145 |
| Normalized Enrichment Score (NES) | -3.1725693 |
| Nominal p-value | 0.0 |
| FDR q-value | 0.0 |
| FWER p-Value | 0.0 |
Table: GSEA Results Summary

  

Fig 1: Enrichment plot: RICHARDS\_NOWAKOWSKI\_SCIENCE\_2017\_MGE\_IPC2\_UPREG      
 Profile of the Running ES Score & Positions of GeneSet Members on the Rank Ordered List

  

| PROBE | GENE SYMBOL | GENE\_TITLE | RANK IN GENE LIST | RANK METRIC SCORE | RUNNING ES | CORE ENRICHMENT || 1 | SOX4 |  |  | 18 | 0.402 | 0.0016 | No |
| 2 | PFN2 |  |  | 46 | 0.303 | 0.0005 | No |
| 3 | H3F3A |  |  | 189 | 0.207 | -0.0214 | No |
| 4 | DCLK2 |  |  | 250 | 0.186 | -0.0295 | No |
| 5 | ZBTB20 |  |  | 275 | 0.180 | -0.0315 | No |
| 6 | UBB |  |  | 335 | 0.169 | -0.0396 | No |
| 7 | KAT2B |  |  | 347 | 0.166 | -0.0395 | No |
| 8 | ABAT |  |  | 435 | 0.145 | -0.0527 | No |
| 9 | GAPDH |  |  | 494 | 0.138 | -0.0610 | No |
| 10 | IDH2 |  |  | 615 | 0.124 | -0.0801 | No |
| 11 | PCBP2 |  |  | 738 | 0.110 | -0.0997 | No |
| 12 | RPS10 |  |  | 862 | 0.099 | -0.1195 | No |
| 13 | SKA2 |  |  | 866 | 0.098 | -0.1189 | No |
| 14 | SOX2 |  |  | 975 | 0.090 | -0.1363 | No |
| 15 | FRMD5 |  |  | 1055 | 0.084 | -0.1488 | No |
| 16 | NDUFS4 |  |  | 1144 | 0.079 | -0.1629 | No |
| 17 | TOP2B |  |  | 1236 | 0.074 | -0.1776 | No |
| 18 | MXI1 |  |  | 1247 | 0.073 | -0.1785 | No |
| 19 | CCDC88A |  |  | 1279 | 0.072 | -0.1829 | No |
| 20 | EPHA4 |  |  | 1342 | 0.069 | -0.1927 | No |
| 21 | APBB2 |  |  | 1358 | 0.068 | -0.1945 | No |
| 22 | SPAG9 |  |  | 1420 | 0.065 | -0.2042 | No |
| 23 | SAP30BP |  |  | 1441 | 0.065 | -0.2068 | No |
| 24 | CLIC4 |  |  | 1521 | 0.061 | -0.2196 | No |
| 25 | ARL4C |  |  | 1553 | 0.060 | -0.2242 | No |
| 26 | COX8A |  |  | 1583 | 0.059 | -0.2285 | No |
| 27 | ATF7IP |  |  | 1598 | 0.059 | -0.2302 | No |
| 28 | NEDD4L |  |  | 1673 | 0.056 | -0.2422 | No |
| 29 | CREB1 |  |  | 1725 | 0.055 | -0.2503 | No |
| 30 | EFS |  |  | 1730 | 0.054 | -0.2503 | No |
| 31 | PCNP |  |  | 1750 | 0.054 | -0.2529 | No |
| 32 | PIP4K2A |  |  | 1764 | 0.053 | -0.2545 | No |
| 33 | ZEB2 |  |  | 2031 | 0.046 | -0.2995 | No |
| 34 | EAF1 |  |  | 2032 | 0.046 | -0.2989 | No |
| 35 | PDLIM3 |  |  | 2070 | 0.045 | -0.3047 | No |
| 36 | ASAP1 |  |  | 2097 | 0.044 | -0.3086 | No |
| 37 | FIGN |  |  | 2217 | 0.041 | -0.3285 | No |
| 38 | RAP2B |  |  | 2271 | 0.040 | -0.3371 | No |
| 39 | PIK3R3 |  |  | 2273 | 0.040 | -0.3368 | No |
| 40 | TMEM126B |  |  | 2306 | 0.039 | -0.3418 | No |
| 41 | CDK6 |  |  | 2322 | 0.039 | -0.3439 | No |
| 42 | MBIP |  |  | 2323 | 0.039 | -0.3435 | No |
| 43 | CCND2 |  |  | 2367 | 0.038 | -0.3504 | No |
| 44 | USP33 |  |  | 2381 | 0.037 | -0.3522 | No |
| 45 | COPS8 |  |  | 2392 | 0.037 | -0.3535 | No |
| 46 | EIF3D |  |  | 2502 | 0.035 | -0.3717 | No |
| 47 | SMARCA2 |  |  | 2714 | 0.030 | -0.4074 | No |
| 48 | OSBPL11 |  |  | 2872 | 0.027 | -0.4339 | No |
| 49 | SPECC1 |  |  | 2875 | 0.027 | -0.4339 | No |
| 50 | STK39 |  |  | 2943 | 0.026 | -0.4450 | No |
| 51 | PSRC1 |  |  | 2990 | 0.025 | -0.4526 | No |
| 52 | ARID1A |  |  | 3044 | 0.024 | -0.4614 | No |
| 53 | MSI2 |  |  | 3242 | 0.020 | -0.4948 | No |
| 54 | TSC22D2 |  |  | 3343 | 0.017 | -0.5117 | No |
| 55 | HDAC2 |  |  | 3407 | 0.015 | -0.5223 | No |
| 56 | RDX |  |  | 3422 | 0.015 | -0.5245 | No |
| 57 | SMARCA5 |  |  | 3518 | 0.011 | -0.5406 | No |
| 58 | CCAR1 |  |  | 3526 | 0.011 | -0.5416 | No |
| 59 | CHD6 |  |  | 3530 | 0.011 | -0.5420 | No |
| 60 | CBX5 |  |  | 3561 | 0.010 | -0.5470 | No |
| 61 | ARHGEF26 |  |  | 3596 | 0.009 | -0.5527 | No |
| 62 | TUBA1B |  |  | 3658 | 0.007 | -0.5631 | No |
| 63 | BRD7 |  |  | 3671 | 0.006 | -0.5651 | No |
| 64 | DPF2 |  |  | 3681 | 0.006 | -0.5665 | No |
| 65 | MAPRE1 |  |  | 3682 | 0.006 | -0.5665 | No |
| 66 | RYBP |  |  | 3761 | 0.002 | -0.5798 | No |
| 67 | LSAMP |  |  | 3779 | 0.001 | -0.5826 | No |
| 68 | H1FX |  |  | 3867 | -0.002 | -0.5975 | No |
| 69 | HERPUD1 |  |  | 3930 | -0.006 | -0.6080 | No |
| 70 | ERH |  |  | 3933 | -0.006 | -0.6083 | No |
| 71 | KIZ |  |  | 3949 | -0.007 | -0.6108 | No |
| 72 | HNRNPA1 |  |  | 4032 | -0.011 | -0.6246 | No |
| 73 | MEIS1 |  |  | 4218 | -0.023 | -0.6560 | No |
| 74 | GABPB1 |  |  | 4231 | -0.024 | -0.6577 | No |
| 75 | TNPO3 |  |  | 4283 | -0.027 | -0.6661 | No |
| 76 | ZDHHC17 |  |  | 4304 | -0.029 | -0.6692 | No |
| 77 | ANAPC5 |  |  | 4335 | -0.031 | -0.6740 | No |
| 78 | SFMBT1 |  |  | 4353 | -0.032 | -0.6765 | No |
| 79 | RALY |  |  | 4356 | -0.032 | -0.6765 | No |
| 80 | GLCCI1 |  |  | 4365 | -0.033 | -0.6775 | No |
| 81 | MAD2L2 |  |  | 4387 | -0.035 | -0.6806 | No |
| 82 | YBX1 |  |  | 4407 | -0.036 | -0.6835 | No |
| 83 | LIN54 |  |  | 4436 | -0.039 | -0.6878 | No |
| 84 | SLC35B4 |  |  | 4478 | -0.043 | -0.6943 | No |
| 85 | ILDR2 |  |  | 4550 | -0.048 | -0.7059 | No |
| 86 | STAG2 |  |  | 4558 | -0.049 | -0.7065 | No |
| 87 | CEP83 |  |  | 4608 | -0.052 | -0.7143 | No |
| 88 | ODF2 |  |  | 4621 | -0.053 | -0.7157 | No |
| 89 | NONO |  |  | 4633 | -0.054 | -0.7170 | No |
| 90 | RAB3IP |  |  | 4665 | -0.057 | -0.7216 | No |
| 91 | OLA1 |  |  | 4718 | -0.061 | -0.7298 | No |
| 92 | G2E3 |  |  | 4722 | -0.062 | -0.7296 | No |
| 93 | HDGF |  |  | 4737 | -0.063 | -0.7312 | No |
| 94 | RMI1 |  |  | 4837 | -0.071 | -0.7473 | No |
| 95 | CDH2 |  |  | 4839 | -0.071 | -0.7467 | No |
| 96 | RGS12 |  |  | 4856 | -0.072 | -0.7486 | No |
| 97 | ZNF704 |  |  | 4861 | -0.072 | -0.7484 | No |
| 98 | FRYL |  |  | 4870 | -0.073 | -0.7489 | No |
| 99 | SPATA5 |  |  | 4889 | -0.074 | -0.7511 | No |
| 100 | NUP155 |  |  | 4920 | -0.077 | -0.7554 | No |
| 101 | HNRNPR |  |  | 4924 | -0.077 | -0.7550 | No |
| 102 | CTDSPL2 |  |  | 4926 | -0.077 | -0.7543 | No |
| 103 | RTTN |  |  | 4955 | -0.079 | -0.7581 | No |
| 104 | PBRM1 |  |  | 4969 | -0.080 | -0.7594 | No |
| 105 | ACYP1 |  |  | 4975 | -0.081 | -0.7593 | No |
| 106 | ADCY3 |  |  | 4985 | -0.081 | -0.7599 | No |
| 107 | RBBP4 |  |  | 4986 | -0.082 | -0.7590 | No |
| 108 | CDK2 |  |  | 5015 | -0.084 | -0.7628 | No |
| 109 | FANCL |  |  | 5025 | -0.084 | -0.7634 | No |
| 110 | SCLT1 |  |  | 5036 | -0.086 | -0.7641 | No |
| 111 | PIGK |  |  | 5038 | -0.086 | -0.7633 | No |
| 112 | TMSB4X |  |  | 5045 | -0.087 | -0.7633 | No |
| 113 | NUP205 |  |  | 5053 | -0.087 | -0.7635 | No |
| 114 | POLA1 |  |  | 5058 | -0.088 | -0.7631 | No |
| 115 | JADE1 |  |  | 5070 | -0.089 | -0.7640 | No |
| 116 | HP1BP3 |  |  | 5074 | -0.089 | -0.7635 | No |
| 117 | LSM3 |  |  | 5075 | -0.089 | -0.7624 | No |
| 118 | TUBB6 |  |  | 5076 | -0.089 | -0.7614 | No |
| 119 | MDK |  |  | 5110 | -0.093 | -0.7659 | No |
| 120 | WDHD1 |  |  | 5120 | -0.093 | -0.7664 | No |
| 121 | SKA3 |  |  | 5136 | -0.095 | -0.7679 | No |
| 122 | GAS2L3 |  |  | 5152 | -0.097 | -0.7693 | No |
| 123 | AASS |  |  | 5154 | -0.097 | -0.7683 | No |
| 124 | LRP6 |  |  | 5164 | -0.098 | -0.7687 | No |
| 125 | PCM1 |  |  | 5177 | -0.099 | -0.7696 | No |
| 126 | TRIM44 |  |  | 5188 | -0.100 | -0.7702 | No |
| 127 | GPC6 |  |  | 5195 | -0.101 | -0.7700 | No |
| 128 | TMEM245 |  |  | 5206 | -0.102 | -0.7706 | No |
| 129 | NUP160 |  |  | 5208 | -0.102 | -0.7695 | No |
| 130 | PLK4 |  |  | 5210 | -0.102 | -0.7685 | No |
| 131 | HMGN1 |  |  | 5223 | -0.103 | -0.7694 | No |
| 132 | SEPT10 |  |  | 5225 | -0.103 | -0.7684 | No |
| 133 | RNASEH2A |  |  | 5241 | -0.105 | -0.7697 | No |
| 134 | DST |  |  | 5242 | -0.105 | -0.7685 | No |
| 135 | ZNF90 |  |  | 5243 | -0.105 | -0.7673 | No |
| 136 | YEATS4 |  |  | 5250 | -0.106 | -0.7671 | No |
| 137 | KIF20A |  |  | 5260 | -0.107 | -0.7674 | No |
| 138 | CENPQ |  |  | 5266 | -0.107 | -0.7670 | No |
| 139 | TMEM123 |  |  | 5280 | -0.108 | -0.7679 | No |
| 140 | RFWD3 |  |  | 5301 | -0.109 | -0.7701 | No |
| 141 | PTPRE |  |  | 5306 | -0.110 | -0.7695 | No |
| 142 | CENPO |  |  | 5321 | -0.111 | -0.7706 | No |
| 143 | ILF2 |  |  | 5331 | -0.112 | -0.7708 | No |
| 144 | UBR7 |  |  | 5340 | -0.112 | -0.7709 | No |
| 145 | CEP192 |  |  | 5351 | -0.114 | -0.7713 | No |
| 146 | SRPK1 |  |  | 5360 | -0.115 | -0.7713 | Yes |
| 147 | C18orf54 |  |  | 5362 | -0.115 | -0.7702 | Yes |
| 148 | CNTRL |  |  | 5363 | -0.115 | -0.7688 | Yes |
| 149 | RCC1 |  |  | 5369 | -0.115 | -0.7684 | Yes |
| 150 | ZNF43 |  |  | 5375 | -0.116 | -0.7679 | Yes |
| 151 | H2AFV |  |  | 5381 | -0.117 | -0.7674 | Yes |
| 152 | C21orf58 |  |  | 5382 | -0.117 | -0.7660 | Yes |
| 153 | WDR76 |  |  | 5388 | -0.117 | -0.7655 | Yes |
| 154 | SAP30 |  |  | 5390 | -0.117 | -0.7643 | Yes |
| 155 | NCAM1 |  |  | 5391 | -0.117 | -0.7630 | Yes |
| 156 | BAZ1B |  |  | 5405 | -0.119 | -0.7638 | Yes |
| 157 | EXO1 |  |  | 5410 | -0.120 | -0.7631 | Yes |
| 158 | HAUS6 |  |  | 5411 | -0.120 | -0.7617 | Yes |
| 159 | ZGRF1 |  |  | 5420 | -0.121 | -0.7617 | Yes |
| 160 | CCDC150 |  |  | 5426 | -0.121 | -0.7611 | Yes |
| 161 | MGME1 |  |  | 5430 | -0.122 | -0.7602 | Yes |
| 162 | RFC3 |  |  | 5450 | -0.124 | -0.7620 | Yes |
| 163 | RBL1 |  |  | 5472 | -0.128 | -0.7641 | Yes |
| 164 | TUBB |  |  | 5482 | -0.129 | -0.7641 | Yes |
| 165 | TMEM209 |  |  | 5485 | -0.130 | -0.7630 | Yes |
| 166 | CENPL |  |  | 5494 | -0.131 | -0.7628 | Yes |
| 167 | RAD21 |  |  | 5496 | -0.131 | -0.7615 | Yes |
| 168 | NEK2 |  |  | 5505 | -0.132 | -0.7613 | Yes |
| 169 | SKA1 |  |  | 5512 | -0.133 | -0.7608 | Yes |
| 170 | NCAPH2 |  |  | 5520 | -0.133 | -0.7604 | Yes |
| 171 | CEP57 |  |  | 5548 | -0.136 | -0.7635 | Yes |
| 172 | CKAP5 |  |  | 5549 | -0.136 | -0.7619 | Yes |
| 173 | TTF1 |  |  | 5552 | -0.136 | -0.7607 | Yes |
| 174 | CCNF |  |  | 5562 | -0.137 | -0.7606 | Yes |
| 175 | ST8SIA5 |  |  | 5567 | -0.138 | -0.7597 | Yes |
| 176 | DEPDC1B |  |  | 5570 | -0.138 | -0.7584 | Yes |
| 177 | BUB3 |  |  | 5583 | -0.141 | -0.7588 | Yes |
| 178 | HMGB3 |  |  | 5584 | -0.142 | -0.7572 | Yes |
| 179 | CENPW |  |  | 5585 | -0.142 | -0.7556 | Yes |
| 180 | PSIP1 |  |  | 5590 | -0.143 | -0.7546 | Yes |
| 181 | NCAPH |  |  | 5595 | -0.144 | -0.7536 | Yes |
| 182 | DCLRE1C |  |  | 5596 | -0.144 | -0.7519 | Yes |
| 183 | AHI1 |  |  | 5598 | -0.144 | -0.7504 | Yes |
| 184 | RIF1 |  |  | 5603 | -0.145 | -0.7494 | Yes |
| 185 | SOX6 |  |  | 5612 | -0.146 | -0.7491 | Yes |
| 186 | RNF168 |  |  | 5623 | -0.147 | -0.7491 | Yes |
| 187 | CENPJ |  |  | 5625 | -0.147 | -0.7476 | Yes |
| 188 | FANCB |  |  | 5631 | -0.149 | -0.7467 | Yes |
| 189 | GEN1 |  |  | 5633 | -0.150 | -0.7451 | Yes |
| 190 | PRIM1 |  |  | 5636 | -0.150 | -0.7437 | Yes |
| 191 | HIRIP3 |  |  | 5638 | -0.151 | -0.7421 | Yes |
| 192 | CDCA8 |  |  | 5642 | -0.151 | -0.7409 | Yes |
| 193 | EXOSC9 |  |  | 5643 | -0.151 | -0.7392 | Yes |
| 194 | MCM6 |  |  | 5644 | -0.151 | -0.7374 | Yes |
| 195 | KIFC1 |  |  | 5645 | -0.152 | -0.7356 | Yes |
| 196 | CASP8AP2 |  |  | 5646 | -0.152 | -0.7339 | Yes |
| 197 | CEP152 |  |  | 5649 | -0.152 | -0.7325 | Yes |
| 198 | TRA2B |  |  | 5652 | -0.152 | -0.7310 | Yes |
| 199 | CLIC1 |  |  | 5664 | -0.154 | -0.7311 | Yes |
| 200 | CEP135 |  |  | 5669 | -0.155 | -0.7300 | Yes |
| 201 | DIAPH1 |  |  | 5671 | -0.155 | -0.7284 | Yes |
| 202 | PARPBP |  |  | 5682 | -0.157 | -0.7283 | Yes |
| 203 | TTK |  |  | 5684 | -0.157 | -0.7266 | Yes |
| 204 | DSN1 |  |  | 5687 | -0.158 | -0.7251 | Yes |
| 205 | MCM8 |  |  | 5701 | -0.161 | -0.7255 | Yes |
| 206 | KNTC1 |  |  | 5703 | -0.161 | -0.7238 | Yes |
| 207 | HNRNPD |  |  | 5705 | -0.161 | -0.7221 | Yes |
| 208 | MPHOSPH9 |  |  | 5706 | -0.161 | -0.7202 | Yes |
| 209 | MSH2 |  |  | 5711 | -0.162 | -0.7190 | Yes |
| 210 | E2F8 |  |  | 5717 | -0.163 | -0.7180 | Yes |
| 211 | CDCA7L |  |  | 5720 | -0.164 | -0.7164 | Yes |
| 212 | NUCKS1 |  |  | 5724 | -0.166 | -0.7150 | Yes |
| 213 | PRIM2 |  |  | 5736 | -0.169 | -0.7149 | Yes |
| 214 | GPSM2 |  |  | 5739 | -0.169 | -0.7133 | Yes |
| 215 | CHD7 |  |  | 5741 | -0.170 | -0.7115 | Yes |
| 216 | EXOSC8 |  |  | 5751 | -0.173 | -0.7110 | Yes |
| 217 | KCNJ2 |  |  | 5756 | -0.174 | -0.7097 | Yes |
| 218 | TOPBP1 |  |  | 5758 | -0.174 | -0.7078 | Yes |
| 219 | TMX1 |  |  | 5759 | -0.174 | -0.7058 | Yes |
| 220 | KIF18B |  |  | 5760 | -0.174 | -0.7038 | Yes |
| 221 | RSRC1 |  |  | 5766 | -0.176 | -0.7026 | Yes |
| 222 | ZNF738 |  |  | 5768 | -0.177 | -0.7007 | Yes |
| 223 | BRIP1 |  |  | 5769 | -0.177 | -0.6987 | Yes |
| 224 | ZNF519 |  |  | 5775 | -0.178 | -0.6975 | Yes |
| 225 | NCAPD2 |  |  | 5778 | -0.179 | -0.6957 | Yes |
| 226 | BARD1 |  |  | 5779 | -0.179 | -0.6937 | Yes |
| 227 | MASTL |  |  | 5781 | -0.179 | -0.6917 | Yes |
| 228 | SMCHD1 |  |  | 5784 | -0.180 | -0.6900 | Yes |
| 229 | CDK5RAP2 |  |  | 5798 | -0.182 | -0.6901 | Yes |
| 230 | HAT1 |  |  | 5803 | -0.184 | -0.6887 | Yes |
| 231 | MIS18A |  |  | 5816 | -0.187 | -0.6885 | Yes |
| 232 | POLQ |  |  | 5818 | -0.187 | -0.6866 | Yes |
| 233 | CCNE2 |  |  | 5827 | -0.190 | -0.6857 | Yes |
| 234 | PSMC3 |  |  | 5831 | -0.190 | -0.6840 | Yes |
| 235 | TIMELESS |  |  | 5832 | -0.190 | -0.6818 | Yes |
| 236 | BLM |  |  | 5833 | -0.191 | -0.6796 | Yes |
| 237 | PRC1 |  |  | 5835 | -0.192 | -0.6775 | Yes |
| 238 | FAM111A |  |  | 5844 | -0.194 | -0.6767 | Yes |
| 239 | FANCD2 |  |  | 5849 | -0.195 | -0.6751 | Yes |
| 240 | CORO1C |  |  | 5851 | -0.195 | -0.6730 | Yes |
| 241 | PTMA |  |  | 5852 | -0.195 | -0.6707 | Yes |
| 242 | RBBP8 |  |  | 5854 | -0.195 | -0.6686 | Yes |
| 243 | PAICS |  |  | 5858 | -0.196 | -0.6669 | Yes |
| 244 | E2F7 |  |  | 5860 | -0.196 | -0.6648 | Yes |
| 245 | CDCA7 |  |  | 5863 | -0.198 | -0.6628 | Yes |
| 246 | CIT |  |  | 5864 | -0.198 | -0.6605 | Yes |
| 247 | HIST1H1E |  |  | 5865 | -0.198 | -0.6582 | Yes |
| 248 | TPM4 |  |  | 5876 | -0.200 | -0.6576 | Yes |
| 249 | CKAP2 |  |  | 5879 | -0.201 | -0.6556 | Yes |
| 250 | SAE1 |  |  | 5888 | -0.203 | -0.6546 | Yes |
| 251 | ANP32B |  |  | 5890 | -0.204 | -0.6524 | Yes |
| 252 | DIAPH3 |  |  | 5893 | -0.205 | -0.6504 | Yes |
| 253 | BTG3 |  |  | 5895 | -0.206 | -0.6482 | Yes |
| 254 | HAUS8 |  |  | 5897 | -0.206 | -0.6460 | Yes |
| 255 | FAT1 |  |  | 5898 | -0.206 | -0.6436 | Yes |
| 256 | SPAG5 |  |  | 5899 | -0.207 | -0.6412 | Yes |
| 257 | BUB1B |  |  | 5901 | -0.207 | -0.6389 | Yes |
| 258 | WEE1 |  |  | 5907 | -0.209 | -0.6374 | Yes |
| 259 | BRCA2 |  |  | 5910 | -0.210 | -0.6353 | Yes |
| 260 | FAM111B |  |  | 5918 | -0.211 | -0.6340 | Yes |
| 261 | PRR11 |  |  | 5926 | -0.214 | -0.6327 | Yes |
| 262 | MMS22L |  |  | 5928 | -0.215 | -0.6304 | Yes |
| 263 | TTF2 |  |  | 5929 | -0.215 | -0.6279 | Yes |
| 264 | NUP107 |  |  | 5949 | -0.220 | -0.6286 | Yes |
| 265 | RACGAP1 |  |  | 5953 | -0.223 | -0.6265 | Yes |
| 266 | ALYREF |  |  | 5955 | -0.225 | -0.6241 | Yes |
| 267 | POLD3 |  |  | 5957 | -0.225 | -0.6217 | Yes |
| 268 | TMEM237 |  |  | 5962 | -0.226 | -0.6197 | Yes |
| 269 | XPO1 |  |  | 5964 | -0.227 | -0.6173 | Yes |
| 270 | NCAPD3 |  |  | 5965 | -0.227 | -0.6146 | Yes |
| 271 | SRSF3 |  |  | 5969 | -0.227 | -0.6125 | Yes |
| 272 | SLC38A1 |  |  | 5972 | -0.228 | -0.6102 | Yes |
| 273 | DTL |  |  | 5975 | -0.229 | -0.6079 | Yes |
| 274 | RTKN2 |  |  | 5977 | -0.229 | -0.6054 | Yes |
| 275 | TMEM106C |  |  | 5979 | -0.230 | -0.6029 | Yes |
| 276 | KIF18A |  |  | 5980 | -0.230 | -0.6002 | Yes |
| 277 | MND1 |  |  | 5982 | -0.232 | -0.5977 | Yes |
| 278 | TROAP |  |  | 5983 | -0.232 | -0.5950 | Yes |
| 279 | MAGI1 |  |  | 5985 | -0.233 | -0.5925 | Yes |
| 280 | USP1 |  |  | 5986 | -0.233 | -0.5898 | Yes |
| 281 | HMGXB4 |  |  | 5991 | -0.235 | -0.5877 | Yes |
| 282 | STMN1 |  |  | 5993 | -0.236 | -0.5852 | Yes |
| 283 | KIF2C |  |  | 5996 | -0.237 | -0.5828 | Yes |
| 284 | SPC24 |  |  | 6000 | -0.238 | -0.5805 | Yes |
| 285 | LIG1 |  |  | 6002 | -0.239 | -0.5779 | Yes |
| 286 | SRSF10 |  |  | 6004 | -0.239 | -0.5753 | Yes |
| 287 | ITGB3BP |  |  | 6005 | -0.240 | -0.5725 | Yes |
| 288 | KIF14 |  |  | 6006 | -0.240 | -0.5697 | Yes |
| 289 | HNRNPU |  |  | 6008 | -0.241 | -0.5671 | Yes |
| 290 | SMC1A |  |  | 6017 | -0.245 | -0.5656 | Yes |
| 291 | CHEK1 |  |  | 6025 | -0.248 | -0.5640 | Yes |
| 292 | HNRNPDL |  |  | 6028 | -0.251 | -0.5614 | Yes |
| 293 | HNRNPA2B1 |  |  | 6029 | -0.251 | -0.5585 | Yes |
| 294 | DEPDC1 |  |  | 6030 | -0.251 | -0.5556 | Yes |
| 295 | VRK1 |  |  | 6034 | -0.256 | -0.5531 | Yes |
| 296 | FEN1 |  |  | 6035 | -0.256 | -0.5501 | Yes |
| 297 | RAD18 |  |  | 6039 | -0.263 | -0.5476 | Yes |
| 298 | SMC3 |  |  | 6041 | -0.265 | -0.5447 | Yes |
| 299 | BUB1 |  |  | 6045 | -0.266 | -0.5421 | Yes |
| 300 | HMGB1 |  |  | 6047 | -0.267 | -0.5392 | Yes |
| 301 | HJURP |  |  | 6055 | -0.271 | -0.5372 | Yes |
| 302 | ARHGAP11A |  |  | 6067 | -0.277 | -0.5359 | Yes |
| 303 | HNRNPH1 |  |  | 6068 | -0.277 | -0.5327 | Yes |
| 304 | MCM5 |  |  | 6069 | -0.278 | -0.5294 | Yes |
| 305 | FOXM1 |  |  | 6070 | -0.279 | -0.5262 | Yes |
| 306 | SPC25 |  |  | 6072 | -0.279 | -0.5231 | Yes |
| 307 | HIST1H1C |  |  | 6077 | -0.284 | -0.5205 | Yes |
| 308 | GMNN |  |  | 6080 | -0.285 | -0.5176 | Yes |
| 309 | FBXO5 |  |  | 6084 | -0.287 | -0.5147 | Yes |
| 310 | SYNE2 |  |  | 6086 | -0.287 | -0.5116 | Yes |
| 311 | KIF22 |  |  | 6089 | -0.290 | -0.5086 | Yes |
| 312 | CENPN |  |  | 6090 | -0.291 | -0.5052 | Yes |
| 313 | DEK |  |  | 6092 | -0.292 | -0.5020 | Yes |
| 314 | NUF2 |  |  | 6093 | -0.293 | -0.4986 | Yes |
| 315 | NCAPG2 |  |  | 6094 | -0.294 | -0.4952 | Yes |
| 316 | RFC2 |  |  | 6095 | -0.294 | -0.4918 | Yes |
| 317 | CDCA5 |  |  | 6099 | -0.296 | -0.4888 | Yes |
| 318 | CCNA2 |  |  | 6100 | -0.297 | -0.4854 | Yes |
| 319 | DUT |  |  | 6107 | -0.303 | -0.4829 | Yes |
| 320 | CKAP2L |  |  | 6108 | -0.303 | -0.4794 | Yes |
| 321 | KIF11 |  |  | 6109 | -0.304 | -0.4758 | Yes |
| 322 | MCM10 |  |  | 6110 | -0.305 | -0.4723 | Yes |
| 323 | MCM3 |  |  | 6113 | -0.307 | -0.4691 | Yes |
| 324 | MELK |  |  | 6117 | -0.312 | -0.4660 | Yes |
| 325 | LBR |  |  | 6118 | -0.312 | -0.4624 | Yes |
| 326 | DSEL |  |  | 6121 | -0.316 | -0.4590 | Yes |
| 327 | MIS18BP1 |  |  | 6122 | -0.316 | -0.4554 | Yes |
| 328 | LMNB1 |  |  | 6134 | -0.325 | -0.4535 | Yes |
| 329 | CDCA2 |  |  | 6135 | -0.326 | -0.4497 | Yes |
| 330 | DDX39A |  |  | 6137 | -0.328 | -0.4461 | Yes |
| 331 | CCNB2 |  |  | 6140 | -0.329 | -0.4426 | Yes |
| 332 | NDC80 |  |  | 6142 | -0.330 | -0.4389 | Yes |
| 333 | AURKB |  |  | 6143 | -0.332 | -0.4351 | Yes |
| 334 | TK1 |  |  | 6144 | -0.334 | -0.4312 | Yes |
| 335 | ARL6IP1 |  |  | 6145 | -0.336 | -0.4273 | Yes |
| 336 | NUDT1 |  |  | 6146 | -0.338 | -0.4234 | Yes |
| 337 | RRM1 |  |  | 6147 | -0.338 | -0.4195 | Yes |
| 338 | CDC20 |  |  | 6154 | -0.344 | -0.4165 | Yes |
| 339 | DLGAP5 |  |  | 6156 | -0.345 | -0.4127 | Yes |
| 340 | KIF15 |  |  | 6158 | -0.346 | -0.4088 | Yes |
| 341 | EZH2 |  |  | 6159 | -0.348 | -0.4048 | Yes |
| 342 | CDKN3 |  |  | 6160 | -0.349 | -0.4007 | Yes |
| 343 | ATAD5 |  |  | 6161 | -0.350 | -0.3967 | Yes |
| 344 | KIF23 |  |  | 6162 | -0.351 | -0.3926 | Yes |
| 345 | HMGN2 |  |  | 6163 | -0.351 | -0.3885 | Yes |
| 346 | KIF4A |  |  | 6164 | -0.355 | -0.3844 | Yes |
| 347 | CDCA3 |  |  | 6165 | -0.355 | -0.3803 | Yes |
| 348 | RAD51AP1 |  |  | 6167 | -0.357 | -0.3763 | Yes |
| 349 | ANP32E |  |  | 6168 | -0.357 | -0.3722 | Yes |
| 350 | CENPK |  |  | 6169 | -0.363 | -0.3680 | Yes |
| 351 | FUS |  |  | 6170 | -0.371 | -0.3637 | Yes |
| 352 | MCM4 |  |  | 6171 | -0.372 | -0.3593 | Yes |
| 353 | KIF20B |  |  | 6172 | -0.373 | -0.3550 | Yes |
| 354 | PCNA |  |  | 6173 | -0.374 | -0.3507 | Yes |
| 355 | DTYMK |  |  | 6177 | -0.381 | -0.3468 | Yes |
| 356 | ECT2 |  |  | 6178 | -0.382 | -0.3423 | Yes |
| 357 | CENPM |  |  | 6179 | -0.386 | -0.3379 | Yes |
| 358 | DBF4 |  |  | 6180 | -0.387 | -0.3334 | Yes |
| 359 | DNMT1 |  |  | 6182 | -0.396 | -0.3289 | Yes |
| 360 | CHAF1A |  |  | 6183 | -0.396 | -0.3243 | Yes |
| 361 | RFC4 |  |  | 6185 | -0.399 | -0.3199 | Yes |
| 362 | NASP |  |  | 6187 | -0.403 | -0.3154 | Yes |
| 363 | MYBL2 |  |  | 6190 | -0.408 | -0.3110 | Yes |
| 364 | GINS2 |  |  | 6194 | -0.420 | -0.3066 | Yes |
| 365 | ZWINT |  |  | 6195 | -0.425 | -0.3017 | Yes |
| 366 | TUBB4B |  |  | 6196 | -0.431 | -0.2967 | Yes |
| 367 | BRCA1 |  |  | 6197 | -0.435 | -0.2916 | Yes |
| 368 | UHRF1 |  |  | 6198 | -0.436 | -0.2866 | Yes |
| 369 | RRM2 |  |  | 6199 | -0.438 | -0.2815 | Yes |
| 370 | DHFR |  |  | 6200 | -0.441 | -0.2764 | Yes |
| 371 | ESCO2 |  |  | 6206 | -0.463 | -0.2719 | Yes |
| 372 | PBK |  |  | 6207 | -0.465 | -0.2665 | Yes |
| 373 | FANCI |  |  | 6209 | -0.469 | -0.2612 | Yes |
| 374 | XRCC2 |  |  | 6212 | -0.482 | -0.2560 | Yes |
| 375 | BIRC5 |  |  | 6214 | -0.484 | -0.2505 | Yes |
| 376 | ANLN |  |  | 6215 | -0.484 | -0.2449 | Yes |
| 377 | HIST1H1A |  |  | 6216 | -0.487 | -0.2392 | Yes |
| 378 | GTSE1 |  |  | 6217 | -0.490 | -0.2336 | Yes |
| 379 | CLSPN |  |  | 6218 | -0.490 | -0.2279 | Yes |
| 380 | SMC4 |  |  | 6219 | -0.490 | -0.2222 | Yes |
| 381 | HELLS |  |  | 6221 | -0.501 | -0.2165 | Yes |
| 382 | TYMS |  |  | 6222 | -0.501 | -0.2107 | Yes |
| 383 | NUSAP1 |  |  | 6223 | -0.507 | -0.2048 | Yes |
| 384 | CENPE |  |  | 6226 | -0.511 | -0.1992 | Yes |
| 385 | NCAPG |  |  | 6227 | -0.513 | -0.1933 | Yes |
| 386 | SMC2 |  |  | 6229 | -0.518 | -0.1875 | Yes |
| 387 | TMPO |  |  | 6233 | -0.522 | -0.1819 | Yes |
| 388 | CKS1B |  |  | 6234 | -0.523 | -0.1758 | Yes |
| 389 | KPNA2 |  |  | 6236 | -0.543 | -0.1697 | Yes |
| 390 | ORC6 |  |  | 6238 | -0.585 | -0.1631 | Yes |
| 391 | CKS2 |  |  | 6239 | -0.585 | -0.1563 | Yes |
| 392 | UBE2T |  |  | 6240 | -0.593 | -0.1494 | Yes |
| 393 | HIST1H1D |  |  | 6241 | -0.601 | -0.1424 | Yes |
| 394 | CCNB1 |  |  | 6242 | -0.606 | -0.1354 | Yes |
| 395 | ATAD2 |  |  | 6243 | -0.607 | -0.1284 | Yes |
| 396 | PTTG1 |  |  | 6245 | -0.622 | -0.1213 | Yes |
| 397 | CDK1 |  |  | 6246 | -0.627 | -0.1140 | Yes |
| 398 | MAD2L1 |  |  | 6247 | -0.628 | -0.1068 | Yes |
| 399 | CENPU |  |  | 6248 | -0.633 | -0.0994 | Yes |
| 400 | HIST1H1B |  |  | 6249 | -0.646 | -0.0919 | Yes |
| 401 | H2AFZ |  |  | 6250 | -0.651 | -0.0844 | Yes |
| 402 | MCM7 |  |  | 6252 | -0.665 | -0.0768 | Yes |
| 403 | TPX2 |  |  | 6253 | -0.667 | -0.0691 | Yes |
| 404 | ASPM |  |  | 6254 | -0.680 | -0.0612 | Yes |
| 405 | UBE2C |  |  | 6255 | -0.750 | -0.0525 | Yes |
| 406 | HMGB2 |  |  | 6258 | -0.766 | -0.0439 | Yes |
| 407 | TOP2A |  |  | 6260 | -0.814 | -0.0347 | Yes |
| 408 | MKI67 |  |  | 6261 | -0.882 | -0.0244 | Yes |
| 409 | HIST1H4C |  |  | 6262 | -1.038 | -0.0124 | Yes |
| 410 | CENPF |  |  | 6263 | -1.068 | 0.0000 | Yes |
Table: GSEA details [plain text format]

  

Fig 2: RICHARDS\_NOWAKOWSKI\_SCIENCE\_2017\_MGE\_IPC2\_UPREG: Random ES distribution      
 Gene set null distribution of ES for **RICHARDS\_NOWAKOWSKI\_SCIENCE\_2017\_MGE\_IPC2\_UPREG**
